# Supplementary material for: Genetic variants affect diurnal glucose levels throughout the day
Source: bioRxiv. 2024 Jul 23:2024.07.22.604631. Preprint. [Version 1] doi: 10.1101/2024.07.22.604631 (PMC11291026; doi:10.1101/2024.07.22.604631)
Supplement: Supplement 2 [file NIHPP2024.07.22.604631v1-supplement-2.pdf]

bioRxiv preprint doi: https://doi.org/10.1101/2024.07.22.604631; this version posted July 23, 2024. The copyright holder for this preprint (which was not certified by peer review) is the author/funder, who has granted bioRxiv a license to display the preprint in perpetuity. It is made available under aCC-BY 4.0 International license.

Supplementary Figures and Tables

Supplementary Figure 1. Association of variants at the *MTNR1B* locus with glucose levels

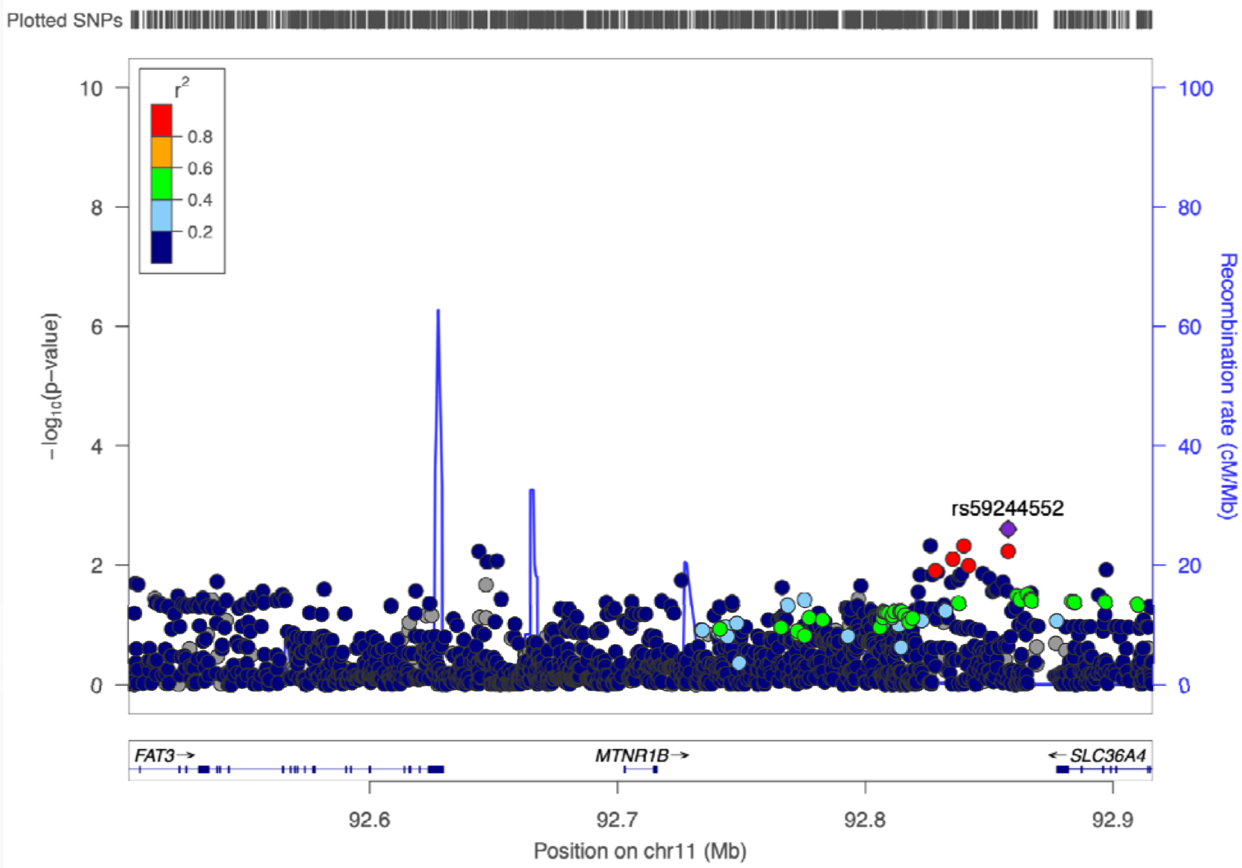

We computed genome-wide association statistics for glucose levels (field id = 30740) in the UKBB. The regional analysis did not show significant association MTNR1B rs10830963 MTNR1B (rs10830963 P = 0.27*) or other variants at the locus.*

bioRxiv preprint doi: https://doi.org/10.1101/2024.07.22.604631; this version posted July 23, 2024. The copyright holder for this preprint (which was not certified by peer review) is the author/funder, who has granted bioRxiv a license to display the preprint in perpetuity. It is made available under aCC-BY 4.0 International license.

Supplementary Figure 2. Association of variants at the *CRY2* locus with glucose levels

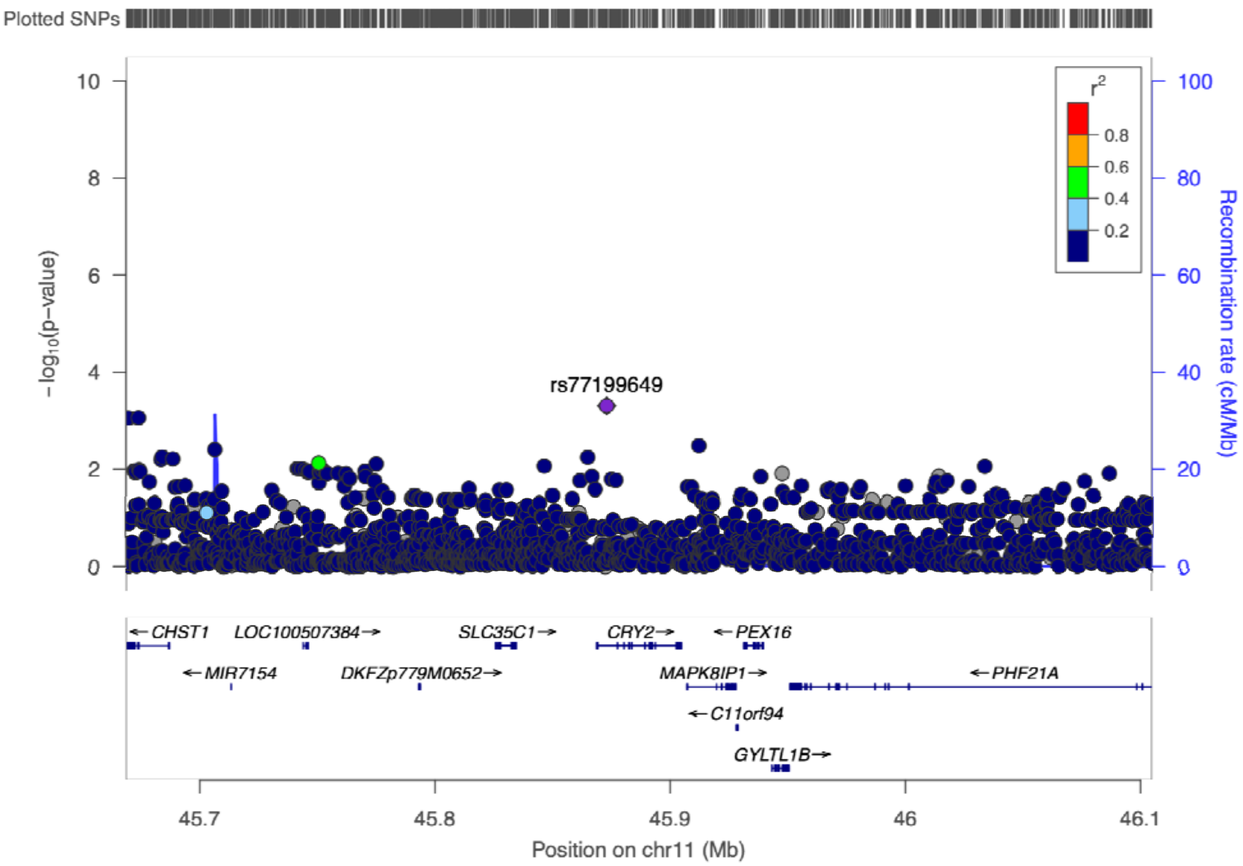

We computed genome-wide association statistics for glucose levels (field id = 30740) in the UKBB. The regional analysis did not show significant association with CRY2 variant (rs12419690 P = 0.46) *or other variants at the locus.*

bioRxiv preprint doi: https://doi.org/10.1101/2024.07.22.604631; this version posted July 23, 2024. The copyright holder for this preprint (which was not certified by peer review) is the author/funder, who has granted bioRxiv a license to display the preprint in perpetuity. It is made available under aCC-BY 4.0 International license.

Supplementary Figure 3. Fasting time stratified by time of sample collection.

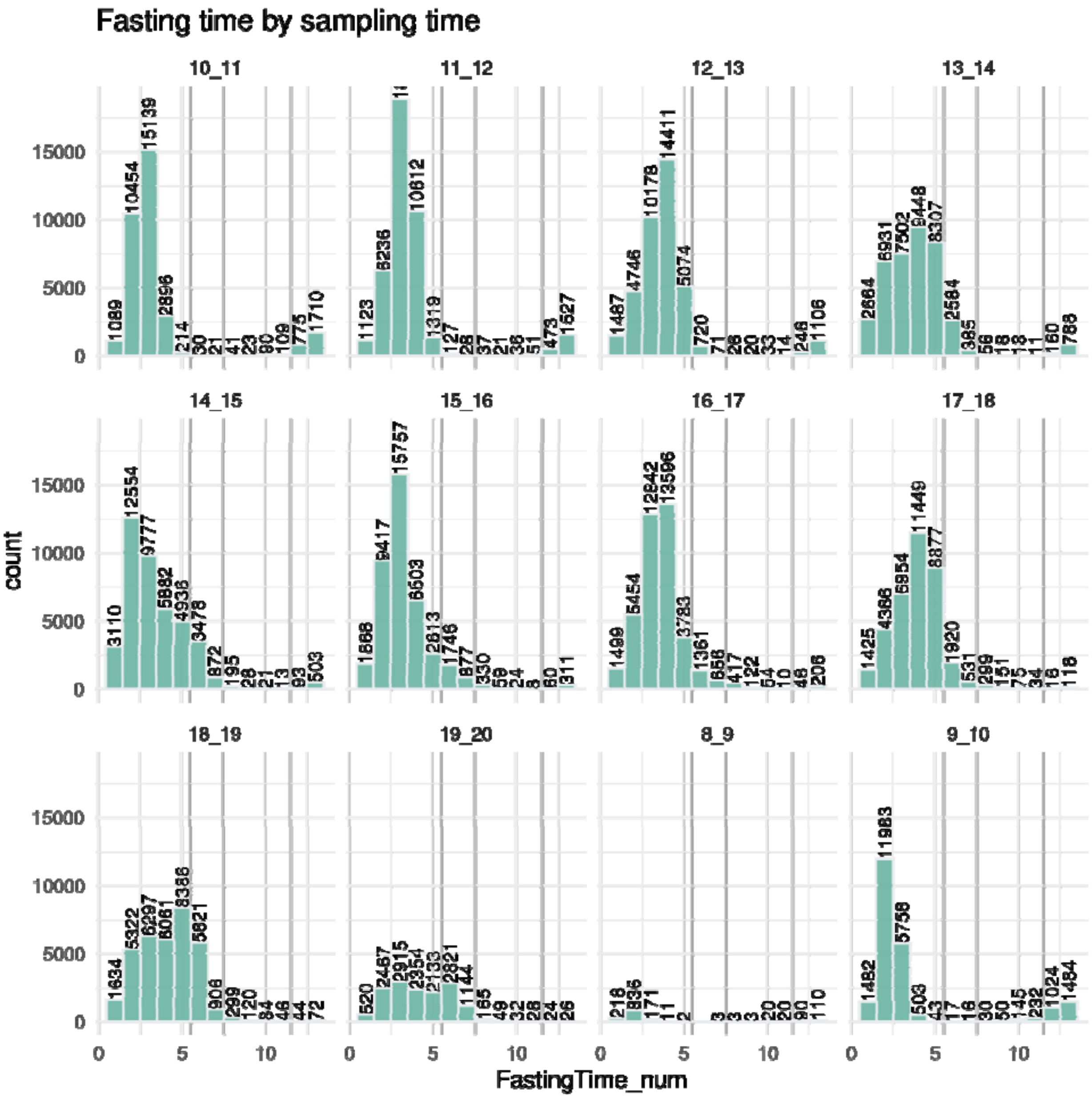

We binned data by time of measurement from 8AM to 8PM, and by number of hours fasting. We then computed the number of individuals in each fasting bin by hours of fasting.

bioRxiv preprint doi: https://doi.org/10.1101/2024.07.22.604631; this version posted July 23, 2024. The copyright holder for this preprint (which was not certified by peer review) is the author/funder, who has granted bioRxiv a license to display the preprint in perpetuity. It is made available under aCC-BY 4.0 International license.

Supplementary Figure 4. Binning process for morning and evening stratification

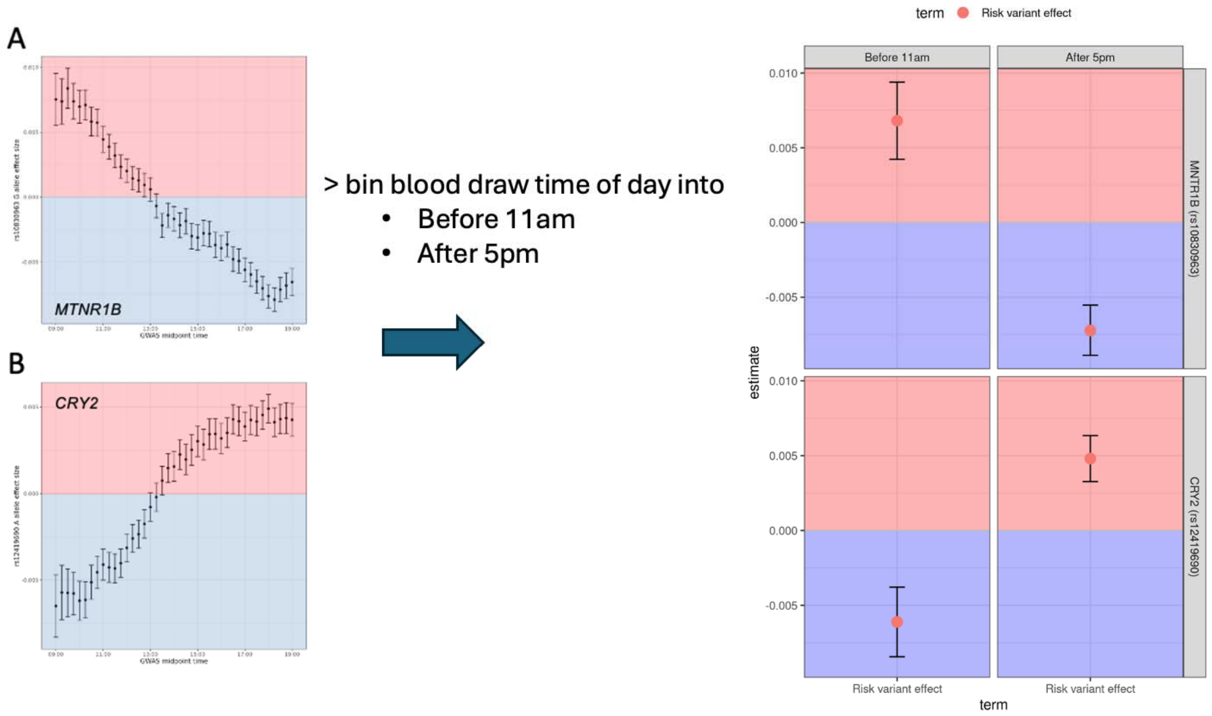

Due to the substantially smaller sample size of individuals fasting in UKBB, we stratified the analysis by sample collected before 11am vs sample collected after 5pm.

Supplementary Table 2. Number of fasting samples at each blood collection time and fasting time.

Fasting time	Blood collection time	n samples
6 hours or more	Before 11am	12,309
6 hours or more	After 5pm	31,441
8 hours or more	Before 11am	12,133
8 hours or more	After 5pm	3,539
12 hours or more	Before 11am	10,573
12 hours or more	After 5pm	607

bioRxiv preprint doi: https://doi.org/10.1101/2024.07.22.604631; this version posted July 23, 2024. The copyright holder for this preprint (which was not certified by peer review) is the author/funder, who has granted bioRxiv a license to display the preprint in perpetuity. It is made available under aCC-BY 4.0 International license.

Supplementary Figure 5. Comparison distribution of association in the morning and evening

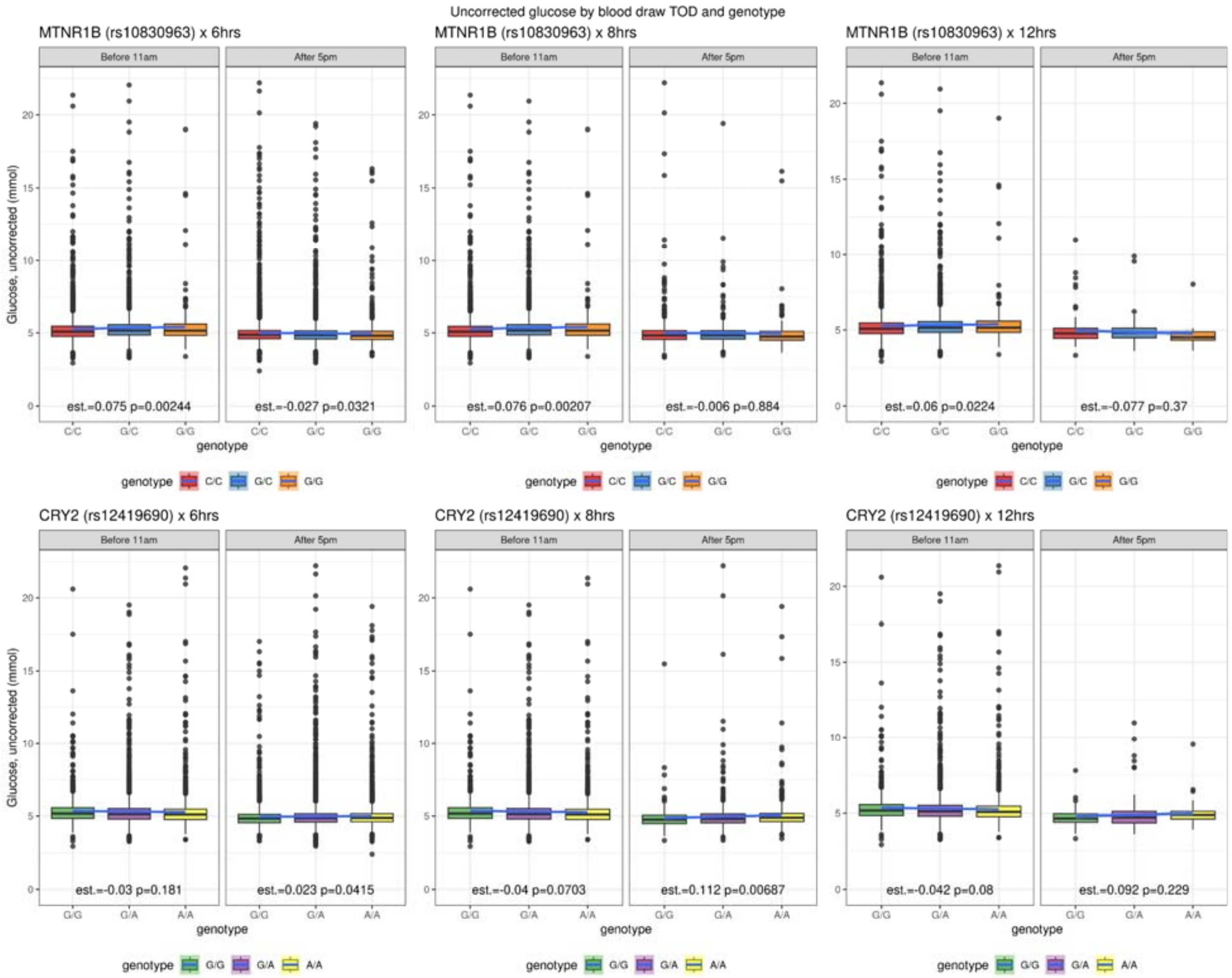

Pairwise comparison of reference and alternative risk alleles in fasting individuals with sample collection time before 11AM or after 5PM with unadjusted glucose levels.

bioRxiv preprint doi: https://doi.org/10.1101/2024.07.22.604631; this version posted July 23, 2024. The copyright holder for this preprint (which was not certified by peer review) is the author/funder, who has granted bioRxiv a license to display the preprint in perpetuity. It is made available under aCC-BY 4.0 International license.

Supplementary Figure 6. Comparison distribution of association of the residual glucose levels in the morning and evening

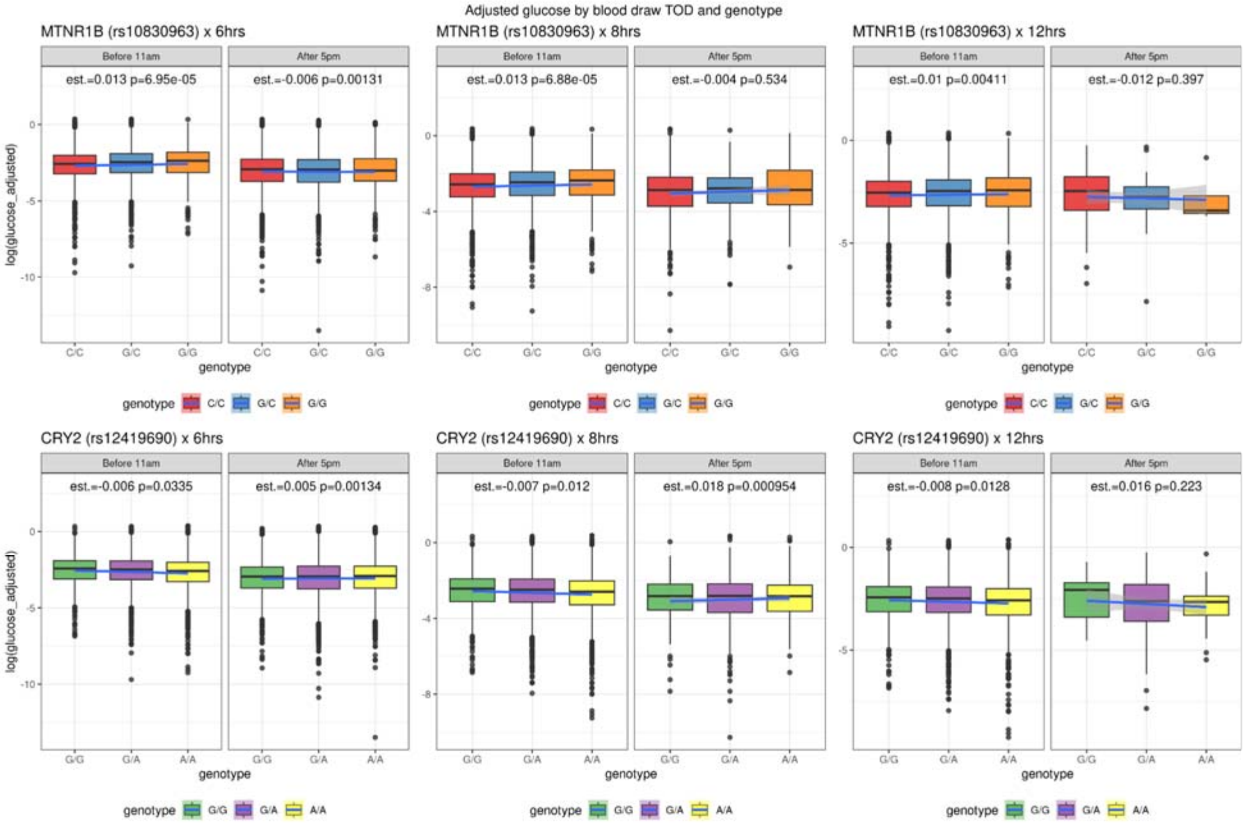

Comparison of reference and alternative risk alleles in fasting individuals with sample collection time before 11AM or after 5PM with the residual glucose levels (see methods).

bioRxiv preprint doi: https://doi.org/10.1101/2024.07.22.604631; this version posted July 23, 2024. The copyright holder for this preprint (which was not certified by peer review) is the author/funder, who has granted bioRxiv a license to display the preprint in perpetuity. It is made available under aCC-BY 4.0 International license.

Supplementary Figure 7. Effect size distribution of *MTNR1B* and *CRY* in Estonian Biobank

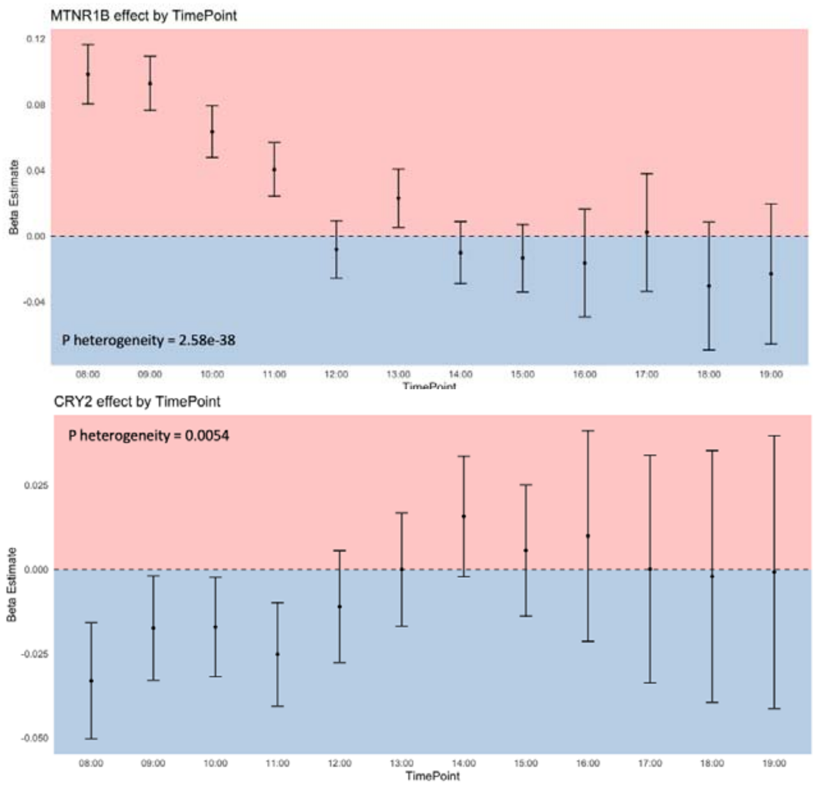

We computed association statistics for each hour of the day in Estonian Biobank and visualize the effect estimate for effect allele stratified by measurement time.

Supplementary Figure 8. Association of *MTNR1B* and *CRY2* loci with chronotype

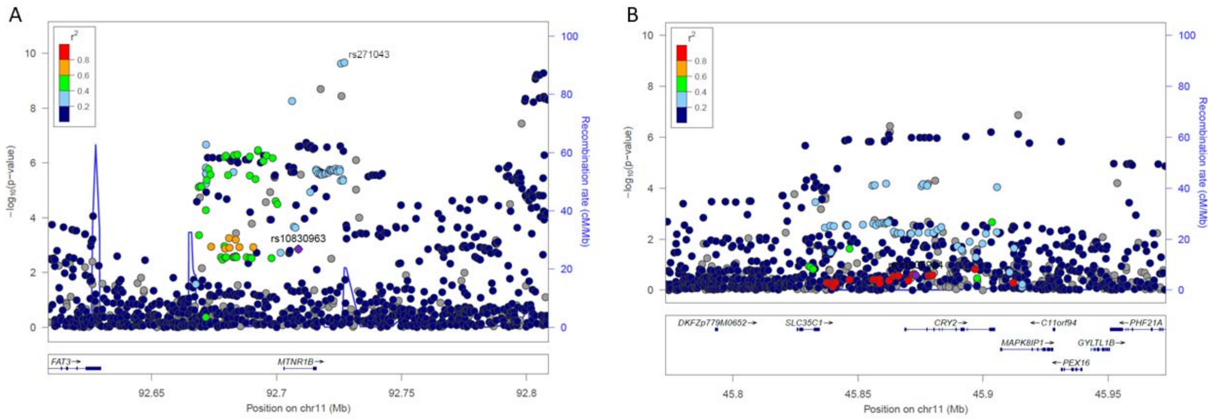

Regional association plot for chronotype at the A) *MTNR1B* and B) *CRY2*. We observed association both at *MTNR1B* and at *CRY2* locus with chronotype. However, the variants that associate with chronotype are not in strong LD (color scale) with the variants that associate with glucose levels.

bioRxiv preprint doi: https://doi.org/10.1101/2024.07.22.604631; this version posted July 23, 2024. The copyright holder for this preprint (which was not certified by peer review) is the author/funder, who has granted bioRxiv a license to display the preprint in perpetuity. It is made available under aCC-BY 4.0 International license.

Supplementary Figure 9. Comparison distribution of association of the residual glucose levels in the morning and evening stratified by insomnia

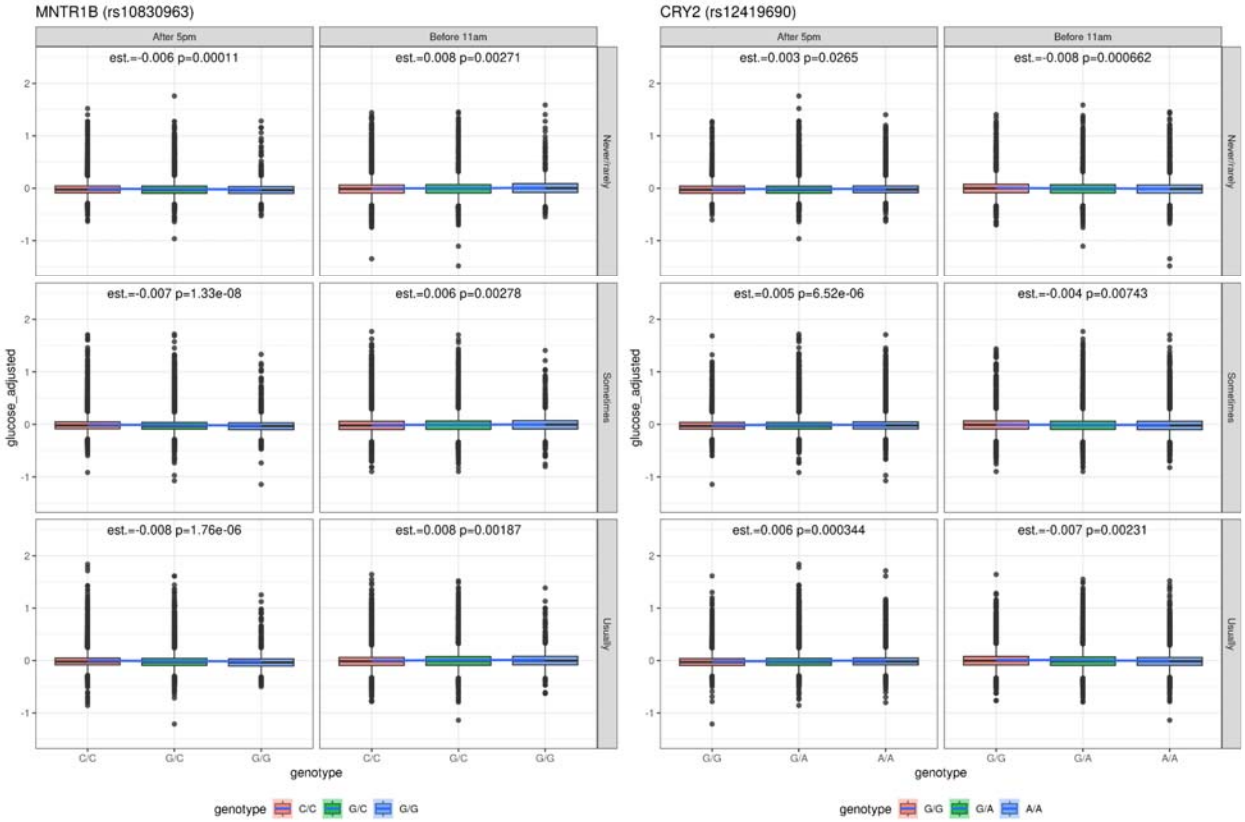

We computed the effect size for *CRY2* and *MTNR1B* effect alleles, stratified by time of day of sampling and insomnia. The estimate reflects additive genotype effect and p-value.

bioRxiv preprint doi: https://doi.org/10.1101/2024.07.22.604631; this version posted July 23, 2024. The copyright holder for this preprint (which was not certified by peer review) is the author/funder, who has granted bioRxiv a license to display the preprint in perpetuity. It is made available under aCC-BY 4.0 International license.

Supplementary Figure 10. Comparison distribution of association of the residual glucose levels in the morning and evening stratified by chronotype

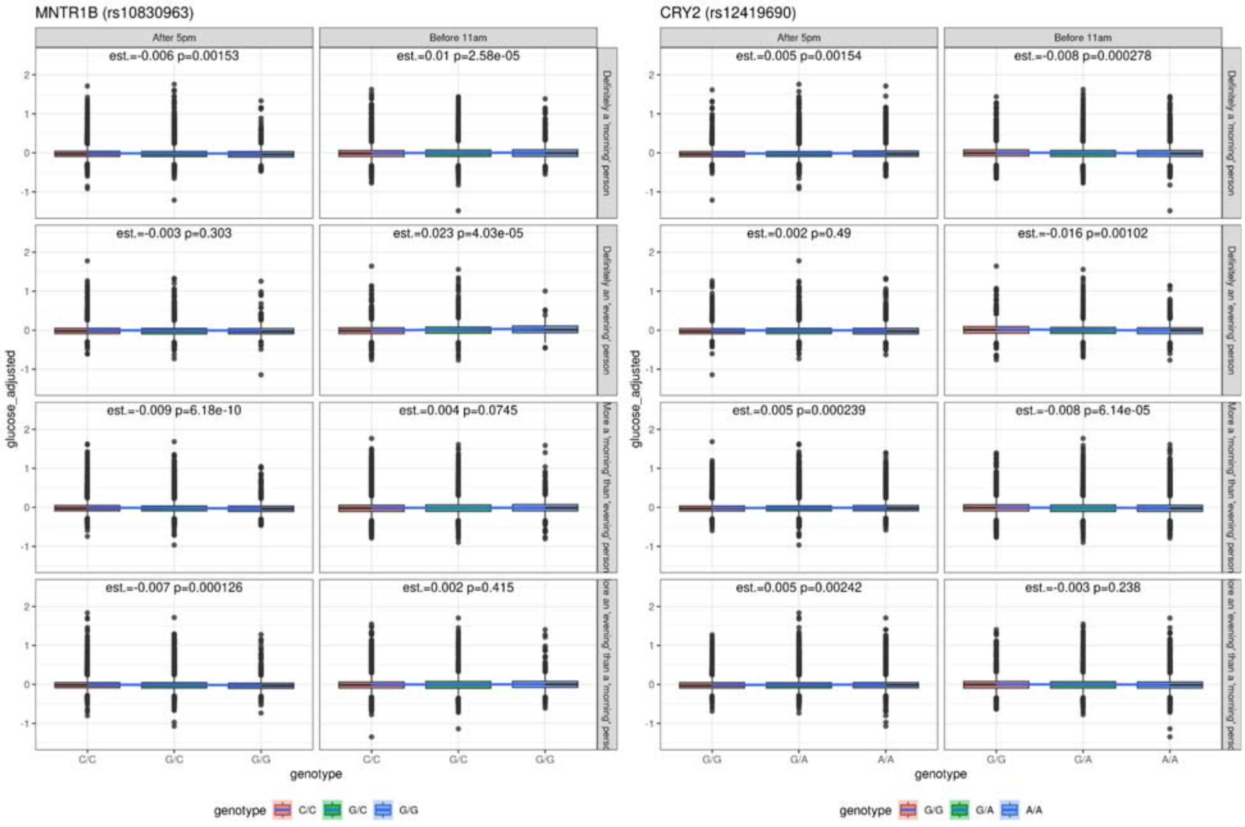

Effect of *CRY2* and *MTNR1B* risk variants, stratified by time of day of sampling and chronotype. The estimate reflects additive genotype effect and p-value.

bioRxiv preprint doi: https://doi.org/10.1101/2024.07.22.604631; this version posted July 23, 2024. The copyright holder for this preprint (which was not certified by peer review) is the author/funder, who has granted bioRxiv a license to display the preprint in perpetuity. It is made available under aCC-BY 4.0 International license.

Supplementary Figure 11. Comparison distribution of association of the residual glucose levels in the morning and evening stratified by sleep duration.

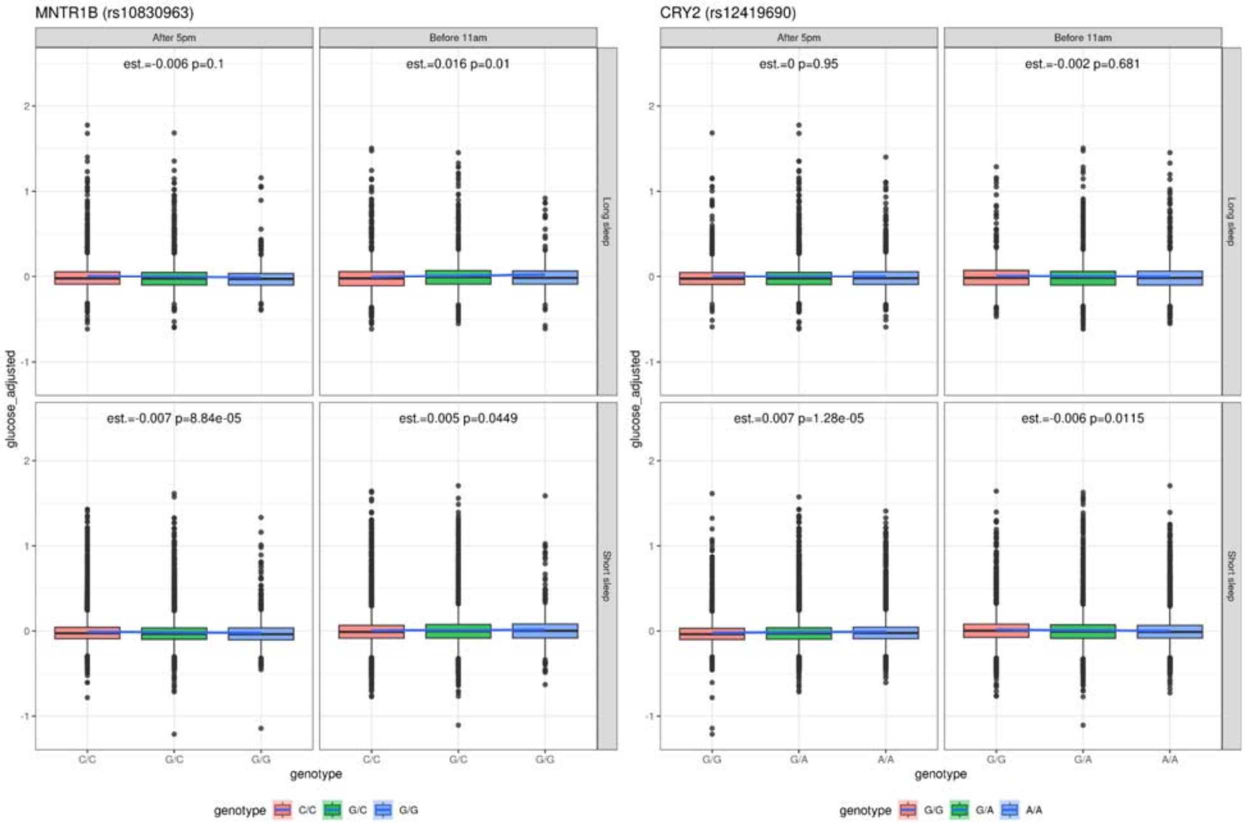

Effect of *CRY2* and *MTNR1B* risk variants, stratified by time of day of sampling and sleep duration. Short sleep was defined as 6 hours or less, and long sleep 9 hours or more. The estimate reflects additive genotype effect and p-value.

bioRxiv preprint doi: https://doi.org/10.1101/2024.07.22.604631; this version posted July 23, 2024. The copyright holder for this preprint (which was not certified by peer review) is the author/funder, who has granted bioRxiv a license to display the preprint in perpetuity. It is made available under aCC-BY 4.0 International license.

Supplementary Figure 12. Comparison distribution of association of the residual glucose levels in the morning and evening stratified by daytime napping/sleeping.

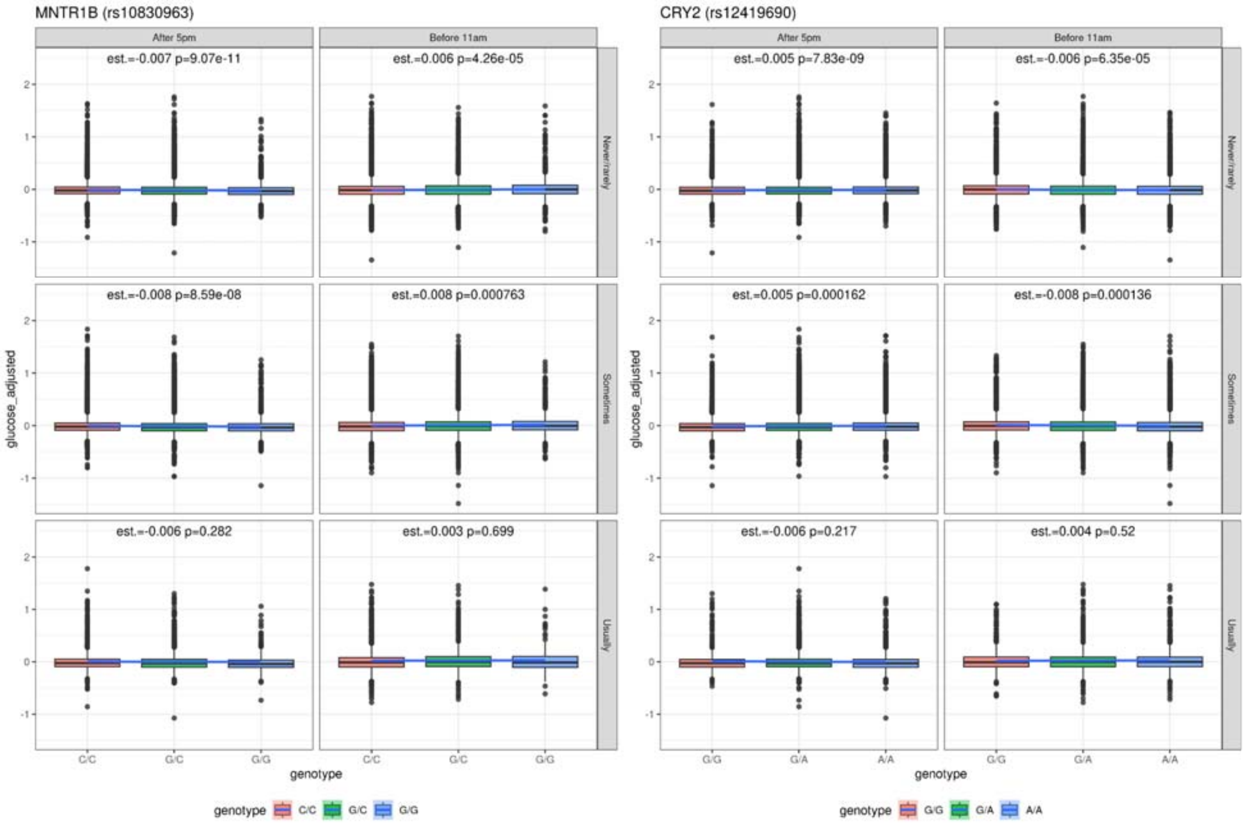

Effect of *CRY2* and *MTNR1B* risk variants, stratified by time of day of sampling and daytime napping/sleeping. The estimate reflects additive genotype effect and p-value.

bioRxiv preprint doi: https://doi.org/10.1101/2024.07.22.604631; this version posted July 23, 2024. The copyright holder for this preprint (which was not certified by peer review) is the author/funder, who has granted bioRxiv a license to display the preprint in perpetuity. It is made available under aCC-BY 4.0 International license.

Supplementary Figure 13. Effect of insomnia, risk genotype and interaction between insomnia and genotype on glucose levels

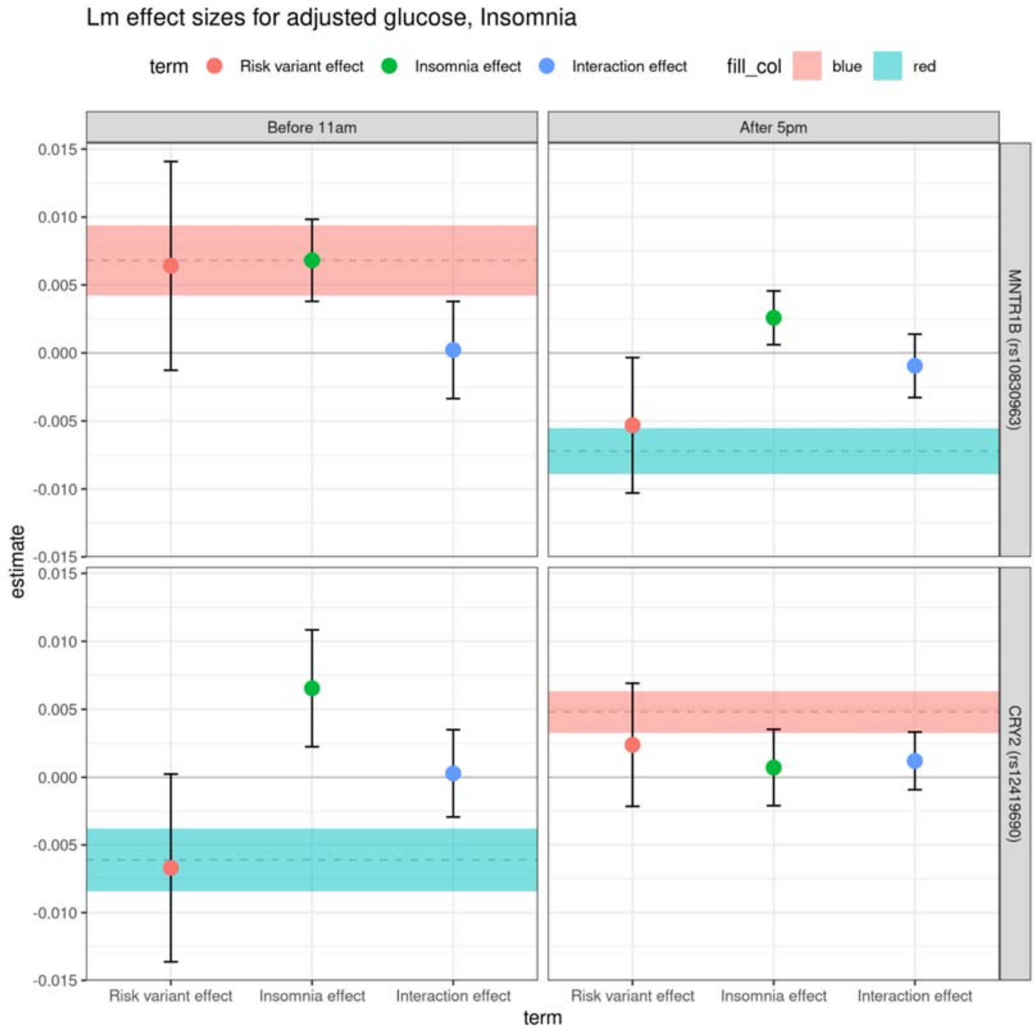

We computed the association of insomnia (green), risk variant (red) and interaction effect (blue) on glucose levels. This shows an effect of insomnia on glucose both in the morning and evening but no interaction between the genotype and insomnia effect. Dashed lines and shaded area in background represent risk variant effect size and 95% confidence intervals over all samples.

bioRxiv preprint doi: https://doi.org/10.1101/2024.07.22.604631; this version posted July 23, 2024. The copyright holder for this preprint (which was not certified by peer review) is the author/funder, who has granted bioRxiv a license to display the preprint in perpetuity. It is made available under aCC-BY 4.0 International license.

Supplementary Figure 14. Effect of napping, risk genotype and interaction between insomnia and genotype on glucose levels

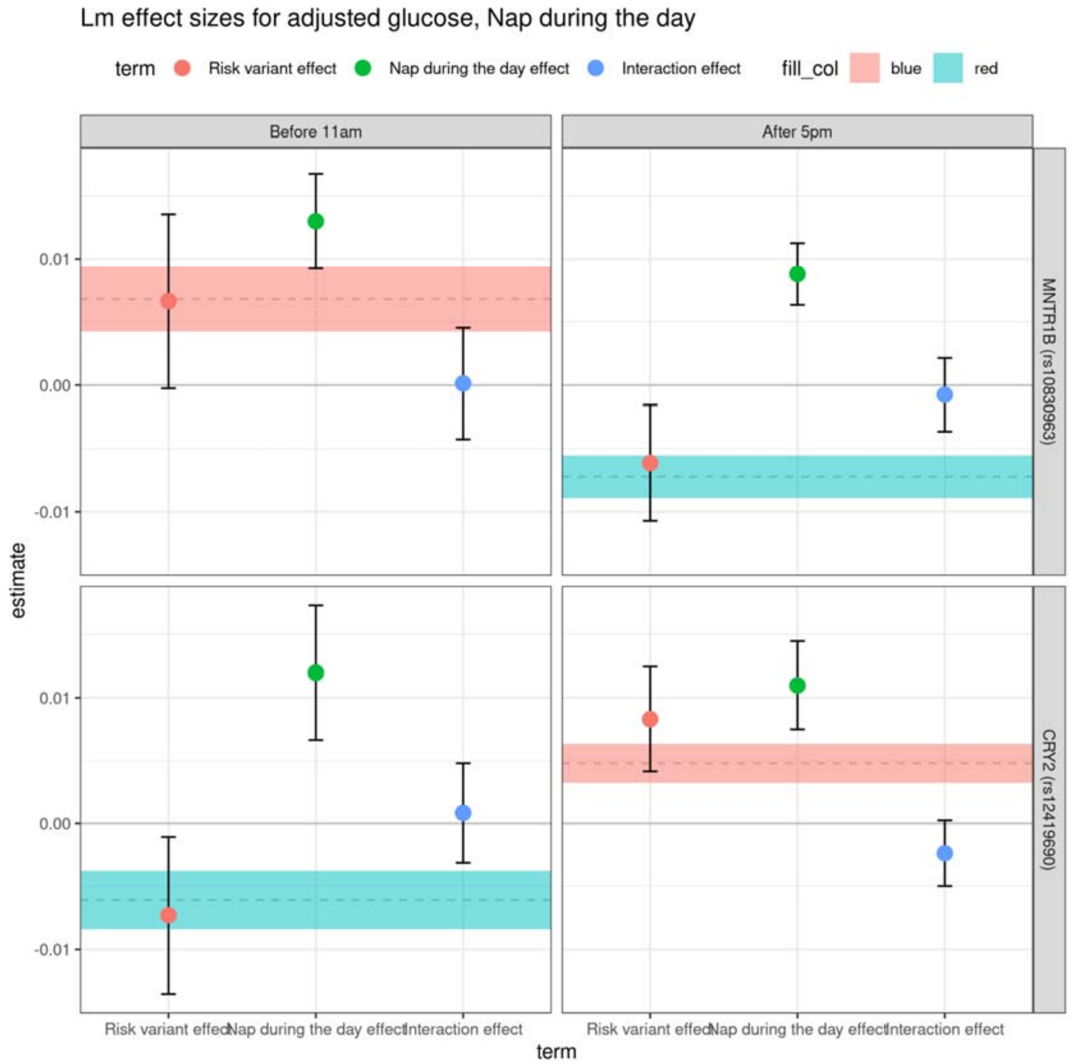

We computed the association of nap during the day (green), risk variant (red) and interaction effect (blue) on glucose levels. This shows an effect of napping on glucose both in the morning and evening but no interaction between the genotype and napping effect. Dashed lines and shaded area in background represent risk variant effect size and 95% confidence intervals over all samples.

bioRxiv preprint doi: https://doi.org/10.1101/2024.07.22.604631; this version posted July 23, 2024. The copyright holder for this preprint (which was not certified by peer review) is the author/funder, who has granted bioRxiv a license to display the preprint in perpetuity. It is made available under aCC-BY 4.0 International license.

Supplementary Figure 15. Effect of dozing, risk genotype and interaction between insomnia and genotype on glucose levels

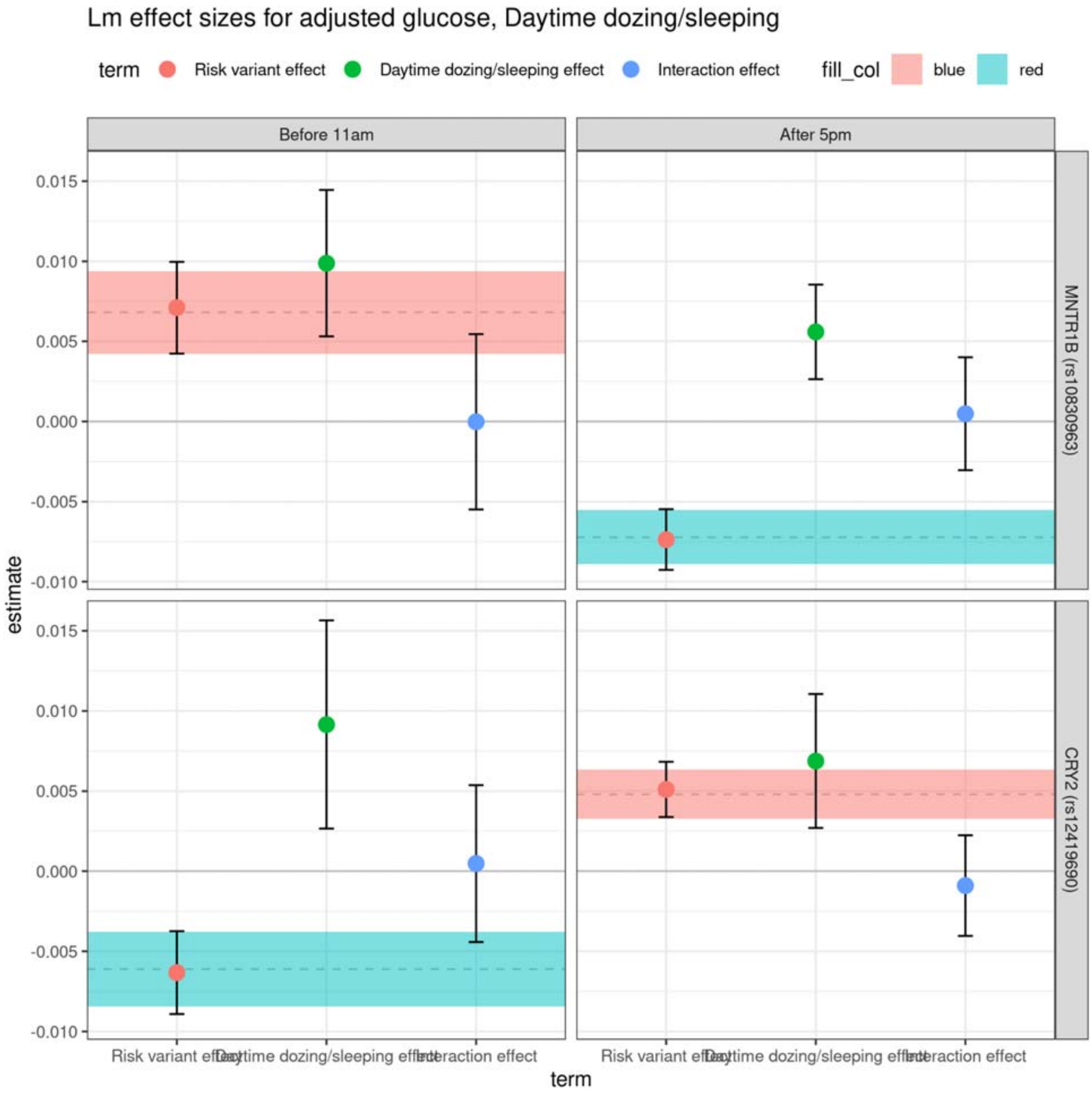

We computed the association of dozing during the day (green), risk variant (red) and interaction effect (blue) on glucose levels. This shows an effect of dozing on glucose both in the morning and evening but no interaction between the genotype and dozing effect. Dashed lines and shaded area in background represent risk variant effect size and 95% confidence intervals over all samples.

bioRxiv preprint doi: https://doi.org/10.1101/2024.07.22.604631; this version posted July 23, 2024. The copyright holder for this preprint (which was not certified by peer review) is the author/funder, who has granted bioRxiv a license to display the preprint in perpetuity. It is made available under aCC-BY 4.0 International license.

Supplementary Figure 16. Effect of sleep duration, risk genotype and interaction between insomnia and genotype on glucose levels

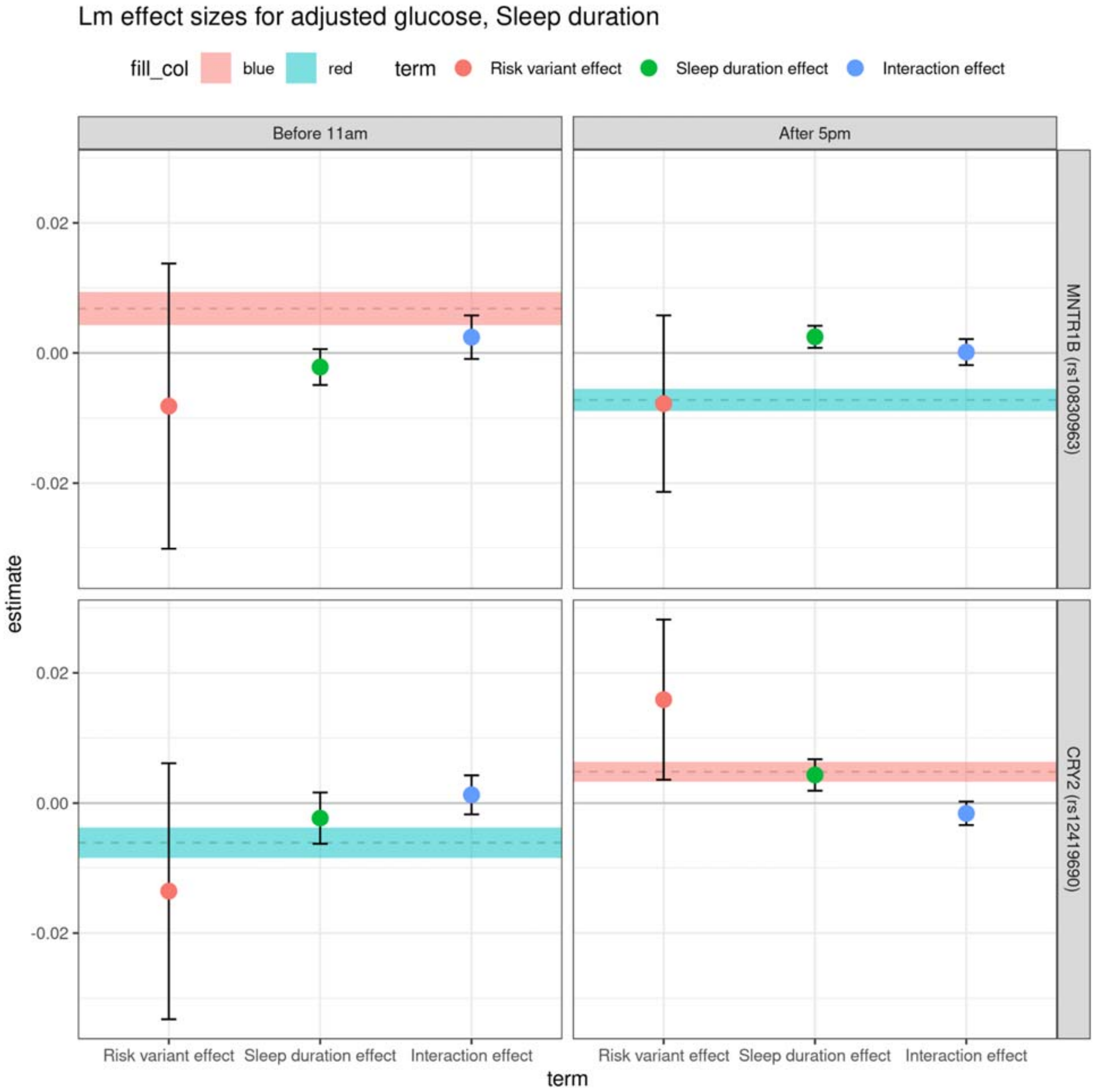

We computed the association of sleep duration (green), risk variant (red) and interaction effect (blue) on glucose levels. Dashed lines and shaded area in background represent risk variant effect size and 95% confidence intervals over all samples.

bioRxiv preprint doi: https://doi.org/10.1101/2024.07.22.604631; this version posted July 23, 2024. The copyright holder for this preprint (which was not certified by peer review) is the author/funder, who has granted bioRxiv a license to display the preprint in perpetuity. It is made available under aCC-BY 4.0 International license.

Supplementary Figure 17. Effect of ease of awakening, risk genotype and interaction between insomnia and genotype on glucose levels

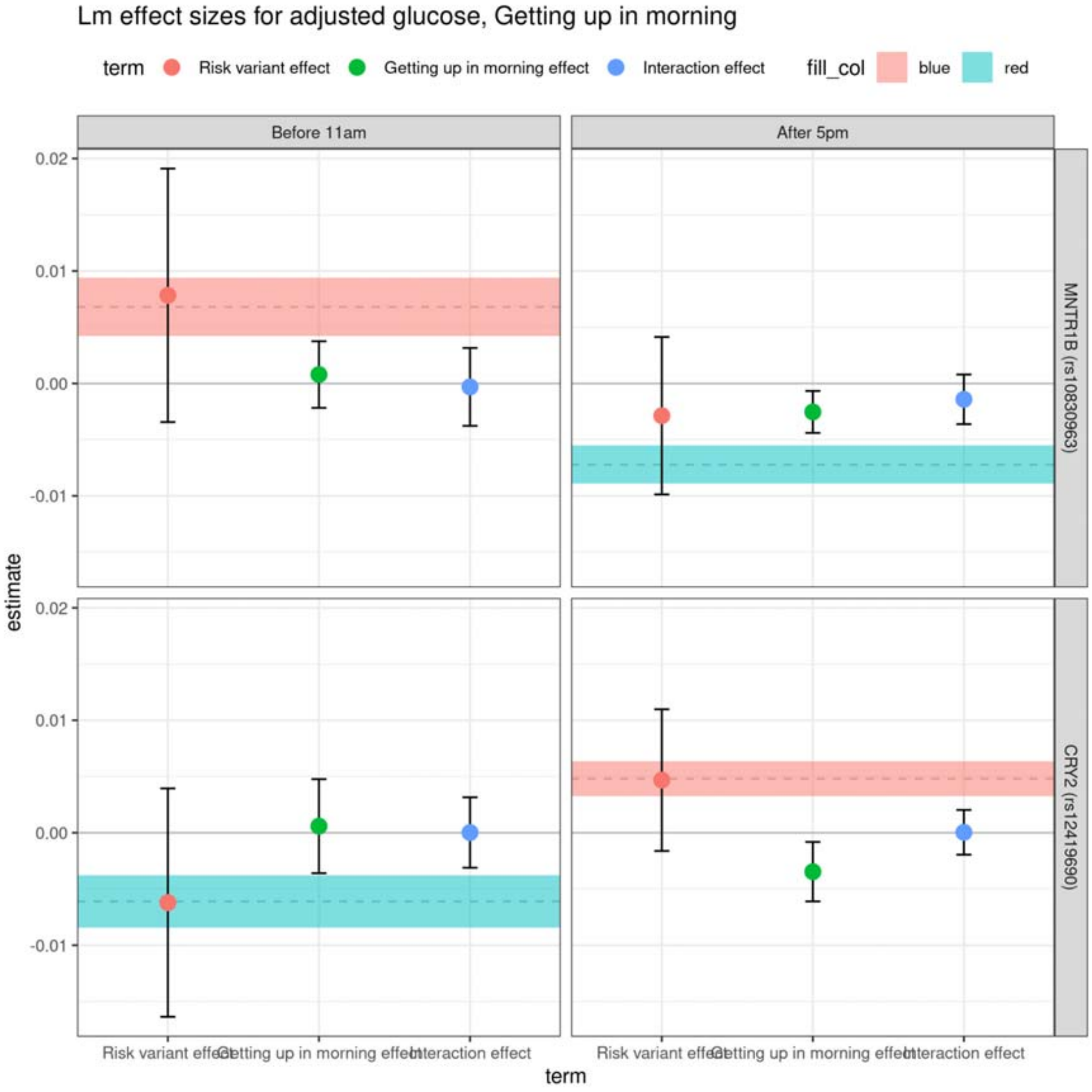

We computed the association of ease of awakening (green), risk variant (red) and interaction effect (blue) on glucose levels. Dashed lines and shaded area in background represent risk variant effect size and 95% confidence intervals over all samples.
